# Bone marrow mesenchymal stem cell‐derived exosomes improve renal fibrosis by reducing the polarisation of M1 and M2 macrophages through the activation of EP2 receptors

**DOI:** 10.1049/nbt2.12071

**Published:** 2021-12-04

**Authors:** Yuqing Lu, Lulu Yang, Xiao Chen, Jing Liu, Anqi Nie, Xiaolan Chen

**Affiliations:** ^1^ Nantong University Nantong Jiangsu China; ^2^ Department of Nephrology The Second People Hospital of Luohe Luohe Henan China

**Keywords:** EP2, exosome, macrophage polarisation, mesenchymal stem cell

## Abstract

Renal fibrosis is the pathological outcome of most end‐stage renal diseases, yet there are still limited therapeutic options for it. In recent years, bone marrow mesenchymal stem cell‐derived exosomes (BM‐MSCs) have received much attention. Here, we investigate the therapeutic effect of BM‐MSCs on unilateral ureteral occlusion (UUO)‐induced interstitial fibrosis in the kidney by modulating prostaglandin E2 receptor 2 (EP2). Renal pathological changes were evident in the UUO group compared to the control group, with significantly increased expression of α‐smooth muscle actin (α‐SMA), fibronectin, Ep2 and F4/80^+^CD86^+^ and F4/80^+^CD206^+^ cells in the UUO group (*p*< 0.05). Pathological changes were alleviated and F4/80^+^CD86^+^ and F480/^+^CD206^+^ cells were reduced after exosome or EP2 agonist intervention compared to the UUO group. These data were further confirmed in vitro. Compared to the lipopolysaccharide (LPS) group and the LPS + exosome + Ah6809 group, the lipopolysaccharide (LPS) + exosome group and the LPS + butaprost group showed a significant decrease in α‐SMA expression, a decrease in the number of F4/80^+^CD86^+^ and F4/80^+^CD206^+^ cells, a decrease in interleukin (IL)‐6 and an increase in IL‐10 levels. Therefore, we conclude that BM‐MSCs can reduce the polarization of M1 and M2 macrophages by activating EP2 receptors, thereby ameliorating renal fibrosis.

**Abbreviations**
AKI: acute kidney injury
BM‐MSCs: bone marrow mesenchymal stem cell‐derived exosomes
Buta: butaprost
CKD: chronic kidney disease
DN: diabetic nephropathy
LPS: lipopolysaccharide
MMT: macrophage myofibroblast transformation
NTA: nanoparticle tracking analysis
PGE2: prostaglandin E2
UUO: unilateral ureteral occlusion
α‐SMA: α‐smooth muscle actin

## 1 INTRODUCTION

Renal interstitial fibrosis is a continuous and progressive process characterised by the activation and migration of myofibroblasts, which, in turn, produce an excessive extracellular matrix and ultimately lead to remodelling of the kidney structure. Although activated myofibroblasts are not the only cell types that are capable of secreting matrix proteins, they are generally considered the most important matrix secreting cells [1]. Myofibroblasts are intermediate phenotypes between fibroblasts and smooth muscle cells. They mainly express α‐smooth muscle actin (α‐SMA), which allows them to connect with the extracellular matrix. Other markers include fibronectin (FN), vimentin etc. [2, 3].

Macrophage infiltration is a common feature of active fibrotic lesions [4]. In chronic kidney disease (CKD) and chronic kidney injury, macrophage infiltration is significantly associated with glomerular sclerosis, interstitial fibrosis, and tubule atrophy [5]. Some studies have shown that the deletion of F4/80^+^ macrophages in the unilateral ureteric obstruction (UUO) kidney can significantly reduce renal fibrosis [6]. Under different pathological conditions, macrophages can be divided into classic activated macrophages (M1) and selectively activated macrophages (M2) [7]. M1 macrophages usually represent the starting point of pro‐fibrosis by releasing pro‐inflammatory mediators, such as interleukin (IL)‐6, which consequently leads to tissue damage, inflammation, and fibrosis. Thus, the inhibition of M1 macrophage polarisation can reduce kidney damage [8]. On the other hand, M2 macrophages release IL‐10 and other anti‐inflammatory mediators to reduce inflammation; but, when the damage persists, M2 cells can be transformed into myofibroblasts to produce the excess extracellular matrix and promote fibrosis [5, 6, 9, 10, 11]. Therefore, the inhibition of M2 macrophage polarisation can reduce renal fibrosis by reducing macrophage myofibroblast transformation (MMT) and extracellular matrix deposition.

Mesenchymal stem cell (MSC)‐derived exosomes are currently considered as a promising treatment approach for kidney diseases. MSC‐derived exosomes contain growth factors, biologically active proteins, lipids, and nucleic acids, which regulate various immune responses [12]. Studies have reported that prostaglandin E2 (PGE2) produced by MSCs can effectively regulate innate and adaptive immunity by inhibiting the inflammatory response and promoting Tregs, which is the key to MSC‐mediated immune regulation. Besides, PGE2 promotes reprogramming of M2 macrophages and increases their IL‐10 production [13]. It has also been reported that MSCs regulate the polarisation of macrophages through PGE2 [14, 15, 16]. It has also been reported that the culture medium of umbilical cord MSCs has an inhibitory effect on renal fibrosis [17]. The aim of this study was to explore the effect of MSC exosomes on renal fibrosis in an UUO model as well as the role and effect of macrophages and EP2 during exosome intervention in renal fibrosis.

## 2 MATERIALS AND METHODS

### 2.1 Isolation and culture of bone marrow MSCs

Two millilitres of fresh bone marrow was collected from healthy volunteers who signed the written informed consent (following the Declaration of Helsinki). Immediately after collection, samples were diluted with 2 ml phosphate‐buffered saline (PBS). The mixture was then placed on the fluid level of lymphocyte separation solution (2 ml, Sigma) and centrifuged at 1000 rpm for 30 min. The liquid was divided into four layers. The second layer (white transparent layer) was aspirated, placed in a cell culture bottle with low‐glucose DMEM medium (Gibco) containing 10% foetal bovine serum (Gibco) and 1% Penicillin‐Streptomycin (Gibco) and cultured in an incubator at 37°C and 5% CO_2_. After 3 days, 4 ml of low‐sugar complete medium was added. Cells were passaged after reaching 80%–90% confluence; the medium was changed every 5 days. After two to three passages, cells were incubated in serum‐free low‐sugar DMEM for 24–48 h (depending on cell density). Consequently, the medium without cells was centrifuged at 3000 rcf for 20 min, and the supernatant was carefully aspirated into a centrifuge tube and frozen at −80°C.

### 2.2 Extraction and identification of exosomes

The supernatant of bone marrow MSCs (BM‐MSCs) from at least three donors was thawed at 4°C and centrifuged (165,000 rcf, 30 min, 4°C). The supernatant was then centrifuged at 150,000 rcf for 2 h at 4°C in an ultracentrifuge to obtain the sediment (exosome). The exosomes were resuspended in PBS. Transmission electron microscopy and nanoparticle tracking analysis (NTA) were used to detect the diameter and morphology of exosomes. Western Blot was used to detect the expression of exosome surface marker proteins CD9, CD63, and the negative expression of surface marker proteins LAMP1.

### 2.3 Establishment of animal models

Fifty Balb/c mice male Balb/c mice (6–8 weeks old, 20 ± 2g) were purchased from the Experimental Animal Center of Medical College of Nantong University. All the animals were housed in an environment with the following conditions: temperature of 22 ± 1°C, relative humidity of 50 ± 1%, and light/dark cycle of 12/12 h. All animal studies (including the mice euthanasia procedure) were done in compliance with the regulations and guidelines of the Nantong University institutional animal care and conducted according to the AAALAC and the IACUC guidelines.

After 2 weeks of acclimatisation, UUO was performed as follows: mice were anesthetised with pentobarbital intraperitoneally (i.p.), and the limbs were fixed and disinfected. The abdominal wall was then cut open (about 5–6 mm to the left of the ventral midline, above the pubic symphysis), and the muscle layer and peritoneum were separated layer by layer to expose the left kidney along with the lower kidney. Next, the left ureter was located, ligated with 40d silk sutures, after which the whole layer was sutured, and the suture was disinfected with 75% alcohol.

Animals experiment was then divided into two parts: Part 1, mice were divided into six groups (five mice/group): control group, UUO0dd group, UUO3d group, UUO7d group, UUO14d group, and UUO21dd group. After each time point (0, 3, 7, 14, and 21 days post‐surgery), mice were euthanised, and kidneys were examined ex vivo.

Part 2: The UUO model was established, and the corresponding drugs were injected according to the following grouping (five mice/group) on the day of modelling: 14d: control group, UUO group, UUO + exosomes group, and UUO + butaprost group. The control group received 0.1 ml PBS and the UUO + exosomes group received 0.1 ml PBS containing 30 μg of exosomes, all of which were injected via the tail vein. In addition, the UUO + butaprost group received an intraperitoneal injection of butaprost (4 mg/kg; Cayman). Mice were sacrificed at days 14 after UUO.

### 2.4 Pathological morphology of the kidney

The kidney was fixed with 4% paraformaldehyde, embedded in paraffin, sectioned and stained with HE, Masson, and Sirius red picric acid staining.

For immunohistochemical analysis, paraffin sections of 3 μm renal tissue were prepared, and the expression of α‐SMA in the renal tissue was detected using an immunohistochemical kit.

Twenty fields were randomly taken from each section, and the degree of renal interstitial matrix deposition was measured. Semi‐quantitative analysis was used to classify the degree of renal interstitial fibrosis: Grade 0: all renal interstitials were normal; Grade 1: <25% of the matrix deposition area in the interstitial; Grade 2: 25%–50%; Grade 3: 50%–75%; and Grade 4: 75%–100%. Renal interstitial fibrosis index was calculated using the following formula: *Renal interstitial fibrosis index* = *[(1* × *n1)* + *(2* × *n2)* + *(3* × *n3)* + *(4* × *n4)]/(n0* + *n1* + *n2* + *n3* + *n4)*, where nx is the number of fields of view with different degrees of fibrosis.

### 2.5 Flow cytometry

The left kidney was collected from each group, washed with physiological saline, and cut into pieces. Tissues were then mixed with trypsin and collagenase and placed in 37°C water bath for digestion. The 100 μm filter was then used to obtain a single‐cell suspension. The suspension was incubated with APC anti‐human CD86 antibody (BioLegend), Alexa Fluor 647 anti‐human F4/80 antibody (BioLegend), PE anti‐human CD206 antibody (BioLegend) at 4°C for 30 min, and then centrifuged at 1500 rpm for 5 min. After washing with PBS, samples were mixed with 300 μl of PBS and transferred to a flow tube at 4°C in the dark.

### 2.6 Western Blot

The protein was extracted from the renal tissue, and denatured by boiling in water for 10 min. Proteins were separated using the following conditions: 80 v for 30 min and then 120 v for 70 min; 300 mA, 80 min transfer membrane. Samples were then blocked for 2 h and incubated with the primary antibody, anti‐prostaglandin E receptor EP2/PTGER2 antibody (Abcam, ab167171), anti‐FN antibody (Abcam, ab1423), recombinant anti‐alpha smooth muscle actin antibody (Abcam, ab124964) at 4°C overnight. Next, samples were incubated with the secondary antibody at room temperature for 1 h. The bands were visualised by chemiluminescence Western blotting detection kit (Millipore, Shanghai, univ).

### 2.7 Macrophage culture

RAW264.7 cells were a kind gift from Professor Yang Bin from the University of Leicester. Cells were cultured in a high‐glucose DMEM medium (Hyclone) containing 10% foetal bovine serum and 1% penicillin‐streptomycin in a humidified atmosphere containing 5%CO_2_/95% air at 37°C. The culture medium was changed every 2 days. Cells were passaged at a density of 60%–70% for at least three generations.

### 2.8 Cell grouping

Macrophage grouping was divided into four parts:

(1) Cells were divided into five groups: control group (0), 0.1 mg/L group, 0.2 mg/L group, 0.5 mg/L group, and 1 mg/L group. Briefly, 1 × 10^5^ cells/well were plated onto a sterilised six‐well plate. After 24 h, different concentrations (0, 0.1, 0.2, 0.5, 1 mg/L) of LPS (Sigma) were added to stimulate the macrophages. Cells were collected after 24 h
(2) Cells were divided into six groups: control group, LPS group, LPS + 0.1 μM group, LPS + 1 μM group, LPS + 10 μM group, and LPS + 20 μM group. Briefly, 1 × 10^5^ cells per well were plated onto a sterilised six‐well plate. After 24 h, all cells, except the control group (blank), were treated with (0.1 mg/L) LPS to stimulate the macrophages. After 1 h, LPS wells were treated with different concentrations (0, 0.1, 1, 10. 20 μM) of butaprost. Cells were collected after 24 h
(3) Cells were divided into six groups: control group, LPS group, LPS + 0.1 μM group, LPS + 1 μM group, LPS + 10 μM group, and LPS + 20 μM group. Briefly, 1 × 10^5^ cells per well were plated onto a sterilised six‐well plate. After 24 h, all cells, except the control group (blank), were treated with (0.1 mg/L) LPS to stimulate the macrophages. After 1 h, LPS wells were treated with different concentrations (0, 0.1, 1, 10. 20 μM) of Ah6809. Cells were collected after 24 h
(4) Cells were divided into five groups: control group, LPS group, LPS + exosome group, LPS + butaprost group, and LPS + exosome + AH6809 group. Briefly, 1 × 10^5^ cells per well were plated onto a sterilised six‐well plate. After 24 h, all cells, except the control group (blank), were treated with (0.1 mg/L) LPS to stimulate the macrophages. After 1 h, LPS wells were treated with the following drugs: LPS group, treated with PBS; LPS + exosome group, treated with 15 μg exosomes; LPS + butaprost group, treated with 20 μM butaprost; LPS + exosome + Ah6809, treated with 15 μg exosomes and 20 μM AH6809. Cells were collected after 24 h

### 2.9 ELISA

After treatment, the cell supernatant was then collected and centrifuged at 3000 rcf/min for 20 min. ELISA kit (Westang) was used to detect IL‐6 and IL‐10 content in the cell supernatant.

### 2.10 Flow cytometer detection

After the macrophages were grouped according to the method described above, they were cultured for 24 h. After trypsinisation, cells were centrifuged at 1500 rpm for 5 min to discard the supernatant. Cells were then mixed with PBS and placed in a flow tube. The flow detection was done as previously described above.

### 2.11 Statistical analysis

The data was analysed using the Graphpad Prism software. For comparison between groups, the normal distribution was analysed by one‐way ANOVA; for the comparison between the two groups, the normal distribution was analysed by *t*‐test. *p* < 0.05 was considered to be statistically significant.

## 3 RESULTS

### 3.1 Isolation and identification of exosomes from BM‐MSCs

**FIGURE 1:**
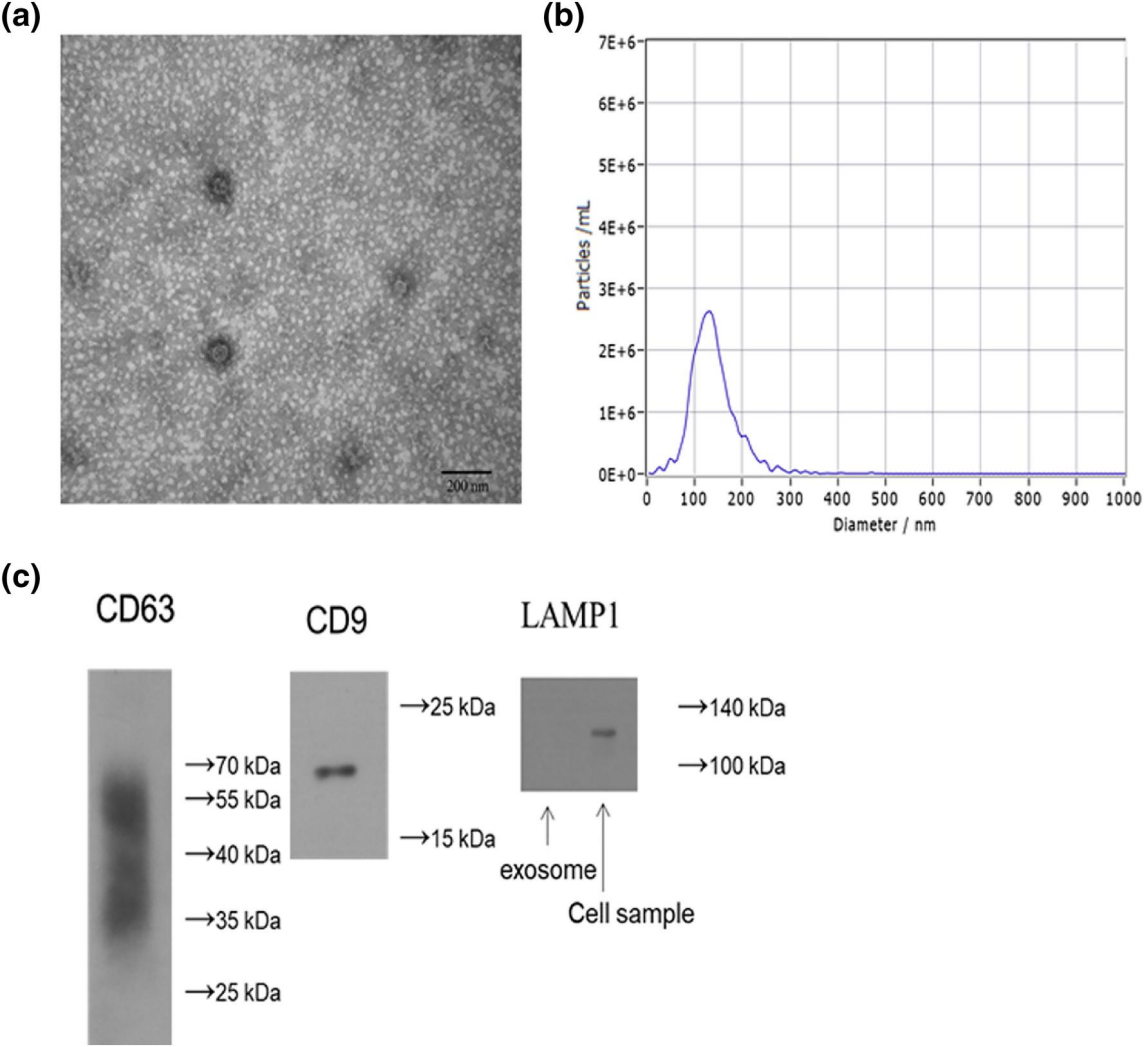
Identification of P3 generation bone marrow mesenchymal stem cell‐derived exosomes and their exosomes. (a) Exosomes observed under a transmission electron microscope. (b) Nanoparticle tracking analysis detects exosomes. (c) Positive and negative expression of exosomal surface marker proteins by Western blot

The exosomes from the P3 generation BM‐MSCs were of uniform size, with consistent membrane vesicles and the double‐layer lipid membrane containing low electron density substances (Figure 1a). Exosomes with a diameter of about 130 nm (range 80‐200 nm) were detected using an NTA (Figure 1b). Western blot showed positive expression of surface marker proteins CD9, CD63, and negative expression of surface marker proteins LAMP1 (Figure 1c).

### 3.2 Pathological changes of UUO mice at different time points

Pathological staining under light microscopy showed that the glomeruli and tubules in the renal tissue of the control group were normal, and there was no inflammation or fibrosis in the renal interstitial. In the UUO3d model group, the renal tubules were dilated; inflammatory cell infiltration was seen in the renal interstitial, and there was no significant extracellular matrix deposition. The UUO7d group showed obvious extracellular matrix deposition and interstitial fibrosis; the UUO14d group had a more significant extracellular matrix deposition and a higher degree of interstitial fibrosis compared with the UUO7d group (*p* < 0.05). Significant structural remodelling occurred in the UUO21d group, but there was no statistical difference in matrix deposition compared with UUO14d (*p* > 0.05).

**FIGURE 2:**
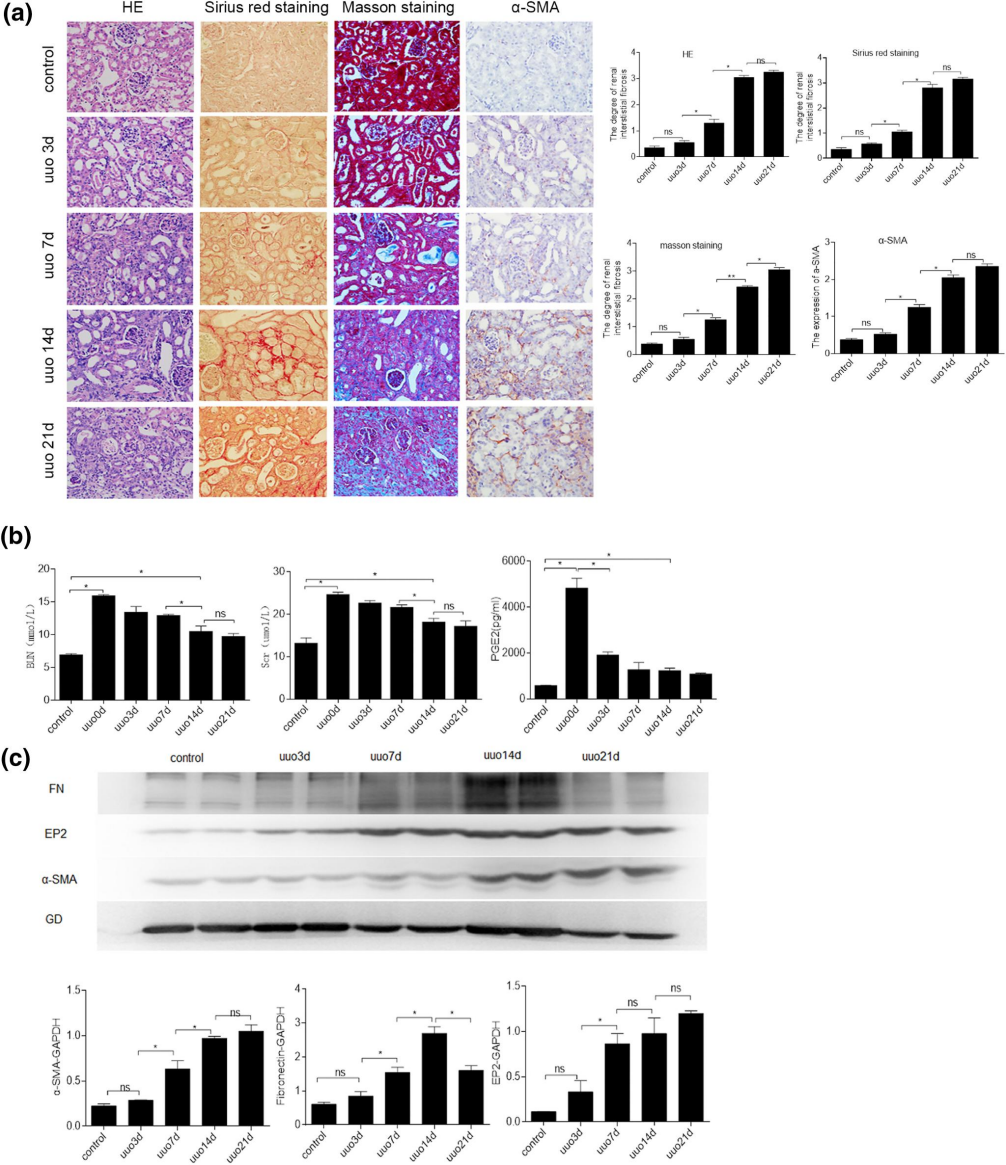
Serum and pathological changes of unilateral ureteral occlusion (UUO) mice at different time points. (a) HE, Masson, Sirius red picric acid staining, and α‐smooth muscle actin (α‐SMA) immunohistochemical staining (X400). (b) Serum Scr, BUN biochemical results, and ELISA detection of prostaglandin E2 levels. (c) Expression of α‐SMA, fibronectin, and EP2 protein levels by Western blot

The results of immunohistochemistry of α‐SMA in renal tissues at different time points showed that there was no significant α‐SMA expression in the kidneys of the control group; α‐SMA expression in the UUO7d group was significantly increased compared with the control group (*p* < 0.05). Compared with the UUO7d group, α‐SMA expression in the UUO14d group was further increased (*p* < 0.05). The UUO21d group had no significant difference in α‐SMA expression compared with the 14‐day model group (*p* > 0.05) (Figure 2a).

### 3.3 Serum and protein changes of UUO mice at different time points

Compared with the control group, the Scr, BUN, and PGE2 are significantly increased in the UUO0d group (*p* < 0.05). Compared with the UUO0d group, significantly lower Scr and BUN levels were observed in the UUO14d and UUO21d groups (*p* < 0.05); however, those levels were still higher compared with the control group (*p* < 0.05) (Figure 2b).

Immunoblotting test results showed no difference in the expression of α‐SMA, FN, and EP2 in the renal tissue between the UUO3d group and the control group (*p* > 0.05). However, the expression significantly increased in the UUO7d group (*p* < 0.05). Compared with the UUO7d group, the expressions of α‐SMA and FN further increased at 14 days (*p* < 0.05), while there was no difference in the expression of EP2 between the UUO14d group and the UUO7d group (*p* > 0.05). The expression of α‐SMA and EP2 in the UUO model group at 21 days was not significantly different from that at 14 days (*p* > 0.05), and the expression of FN was reduced compared to 14 days (*p* < 0.05) (Figure 2c).

### 3.4 The pathological change of renal tissue in different intervention groups

The pathological results of the renal tissue in different intervention groups showed that the glomeruli and tubules in the renal tissue of the control group were normal, and the renal interstitial tissue had no obvious inflammation and no stroma deposition. Compared with the control group, the UUO model group had dilated tubules, infiltrated renal interstitial inflammatory cells, and increased extracellular matrix deposition. Compared with the UUO group, the exosomes or Butaprost significantly reduced interstitial matrix deposition in UUO mice (*p* < 0.05), and there was no difference between the UUO + exosomes group and the UUO + butaprost group (*p* > 0.05).

**FIGURE 3:**
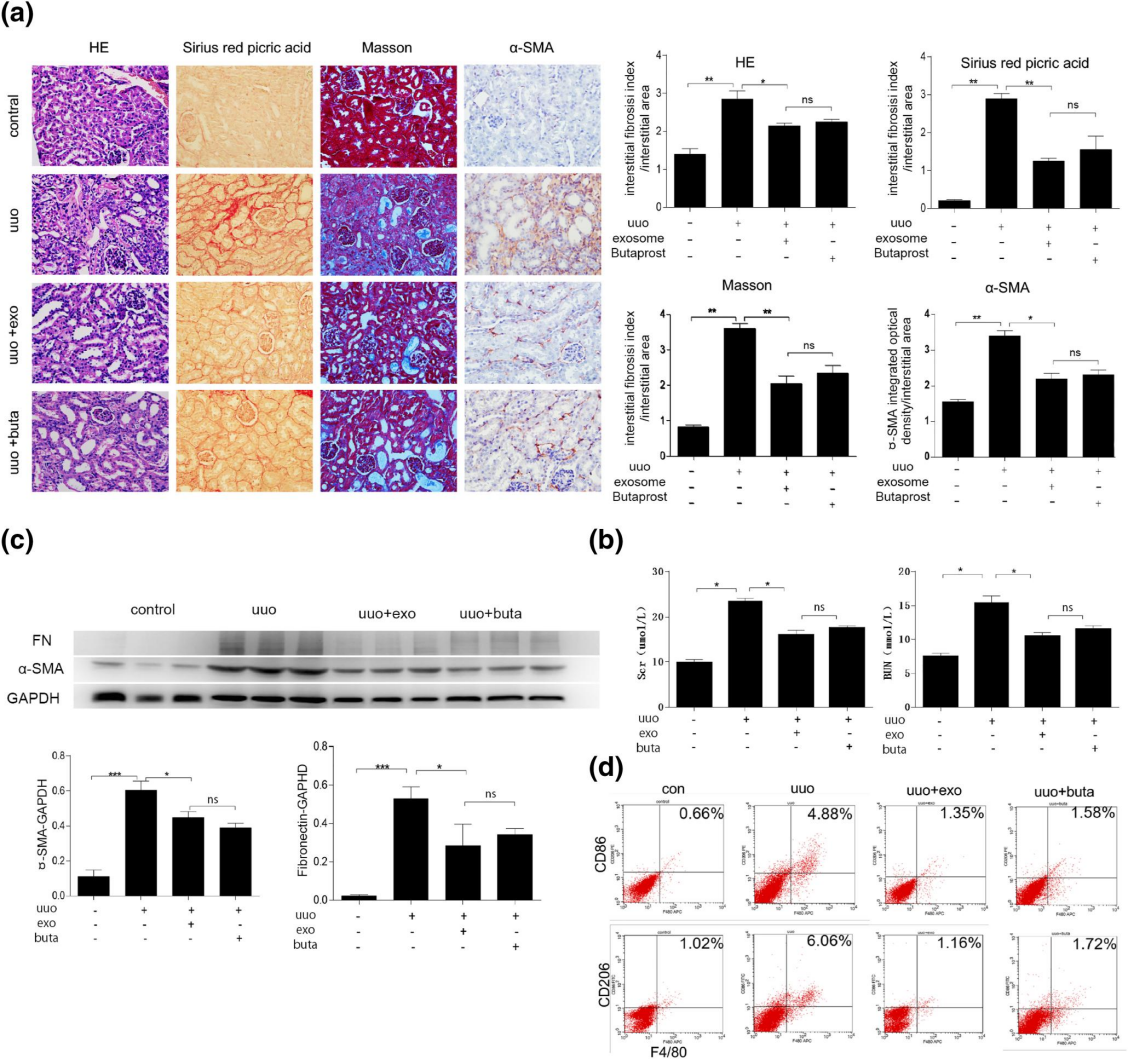
Exosomes and EP2 agonists reduce macrophage polarisation and matrix deposition in unilateral ureteral occlusion (UUO) kidneys. (a) HE, Masson, Sirius red picric acid staining to detect pathological changes in renal tissues in different intervention groups. (b) Serum biochemical changes in different intervention groups. (c) Western detection of α‐smooth muscle actin (α‐SMA) and Fibronectin protein expression in renal tissues of different intervention groups. (d) Flow cytometry to detect the number of F4/80^+^CD86^+^ and F4/80^+^CD206^+^ macrophages in the renal tissue of different intervention groups

No expression of α‐SMA was found in the control group. Compared with the control group, the expression of α‐SMA was significantly increased in the UUO group (*p* < 0.05). The expression of α‐SMA significantly decreased after treating UUO mice with exosomes and Butaprost (*p* < 0.05), and there was no difference between the UUO + exosomes group and the UUO + butaprost group (*p* > 0.05) (Figure 3a).

### 3.5 The serum change in different intervention groups

The serum biochemical results of different intervention groups showed that Scr and BUN in the UUO group were significantly higher than the control group, and the difference was statistically significant (*p* < 0.05). The levels of Scr and BUN in the UUO + exosomes group and the UUO + butaprost group were significantly lower than those in the UUO group (*p* < 0.05). At the same time, there was no difference between the UUO + exosomes group aand the UUO + butaprost group (*p* > 0.05) (Figure 3b).

### 3.6 The level of protein change in different intervention groups

Western results of renal tissues in different intervention groups showed that compared with the control group, the expressions of α‐SMA and FN protein in renal tissues were significantly increased (*p* < 0.05). Compared with the UUO group, the expression of α‐SMA and FN in the exosome intervention group and Butaprost intervention group was significantly reduced, and the difference was statistically significant (*p* < 0.05). There was no difference between the UUO + exosomes group and the UUO + butaprost group (*p* > 0.05) (Figure 3c).

### 3.7 The macrophage polarisation change in different intervention groups

The results of flow cytometry of kidney tissues in different intervention groups showed that compared with the control group, the number of F4/80^+^CD86^+^ and F480/^+^CD206^+^ cells in the obstructed kidney of the UUO group was significantly increased (*p* < 0.05). Compared with the UUO group, the number of F4/80^+^CD86^+^ and F480/^+^CD206^+^ macrophages in the exosomal intervention group and Butaprost intervention group were significantly reduced (*p* < 0.05), and there was no difference between the UUO + exosomes group and the UUO + butaprost group (*p* > 0.05) (Figure 3d).

### 3.8 In vitro expression of α‐SMA protein in macrophages at different concentrations of LPS

**FIGURE 4:**
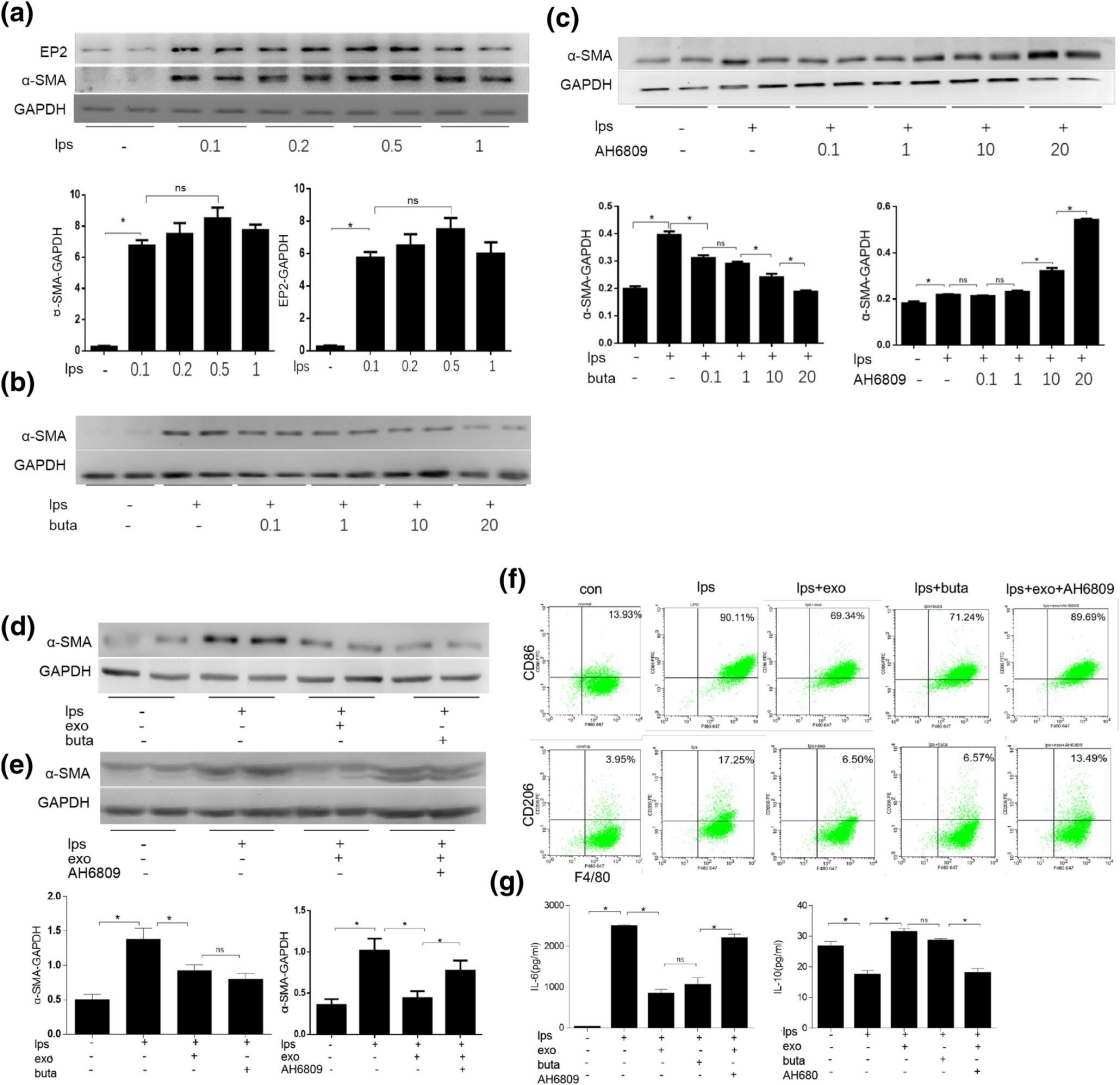
Exosomes activate EP2 receptors to reduce macrophage polarisation. (a) In vitro expression of α‐smooth muscle actin (α‐SMA) protein in macrophages at different concentrations of lipopolysaccharide (LPS). (b) The effect of different concentrations of butaprost on LPS (0.1 μM)‐stimulated macrophages and expression of α‐SMA. (c) The effect of different concentrations of AH6809 on LPS (0.1 μM)‐stimulated macrophages and expression of α‐SMA. (d, e) α‐SMA expression levels of macrophages in different intervention groups. (f) F4/80^+^CD86^+^ and F4/80^+^CD206^+^ expression of macrophages in different intervention groups by flow cytometry. (g) The levels of interleukin (IL)‐6 and IL‐10 in the supernatant of macrophages of different intervention groups were detected by ELISA

Compared with the control group (0 mg/L), the expression of α‐SMA and EP2 protein was significantly increased in the 0.1 mg/L group (*p* < 0.05), while there was no difference among the 0.1 mg/L group, 0.2 group, 0.5 group, and 1 mg/L group (*p* > 0.05) (Figure 4a).

### 3.9 Interfering with different concentrations of Butaprost and AH6809 on LPS‐stimulated macrophages

Butaprost at different concentrations interfered with LPS‐stimulated macrophages and observed the effect on α‐SMA expression. Western blot results showed that compared with the control group, the expression of α‐SMA in the LPS group was significantly increased (*p* < 0.05). Compared with the LPS stimulation group, the expression of α‐SMA in the LPS + 0.1 μM group was significantly reduced (*p* < 0.05). There was no statistically significant difference between the LPS + 0.1 μM group and LPS + 1 μM group. With the increase of concentration, α‐SMA expression continued to decrease, and the difference was statistically significant (*p* < 0.05) (Figure 4b). Therefore, 20 μM Butaprost concentration was used for subsequent experiments.

Compared with the LPS group, the expression of α‐SMA did not significantly change for the LPS + 0.1 μM group and LPS + 1 μM group after the AH6809 intervention. The expression increased in the LPS + 10 μM group, and the difference was statistically significant (*p* < 0.05). The expression of α‐SMA further increased in the LPS + 20 μM group (*p* < 0.05) (Figure 4c).

### 3.10 The α‐SMA expression levels of macrophages in different intervention groups

Compared with the control group, the expression of α‐SMA was significantly increased in the LPS group, while it was decreased in the UUO + exosome group and the UUO + Butaprost group (all *p* < 0.05). There was no statistically significant difference between the UUO + exosome group and the UUO + Butaprost group (*p* > 0.05) (Figure 4d). Compared with the UUO + exosome group, the expression of α‐SMA in the UUO + exosomes + AH6809 group was significantly increased (*p* < 0.05) (Figure 4e).

### 3.11 The F4/80^+^CD86^+^, F4/80^+^CD206^+^ expression of macrophages in different intervention groups

The number of F4/80^+^CD86^+^ and F480/^+^CD206^+^ cells was significantly increased in the LPS group compared with the control group (*p* < 0.05). Compared with the LPS group, the number of F4/80^+^CD86^+^ and F480/^+^CD206^+^ cells in the UUO + Butaprost group and UUO + exosome group was significantly reduced (*p* < 0.05); there was no significant difference between the UUO + exosome group and UUO + Butaprost group (*p* > 0.05). Compared with UUO + exosomes group, the number of F4/80^+^CD86^+^ and F480/^+^CD206^+^ cells in the UUO + exosomes + AH6809 group was significantly increased (*p* < 0.05) (Figure 4f).

### 3.12 The levels of IL‐6 and IL‐10 in the supernatant of macrophages of different intervention groups

Compared with the control group, the levels of IL‐6 in macrophage supernatants in the LPS group were significantly increased (*p* < 0.05), while IL‐10 was significantly reduced (*p* < 0.05). Compared with the LPS group, the levels of IL‐6 in the UUO + exosome group and UUO + Butaprost group were significantly reduced (*p* < 0.05), while the IL‐10 level was significantly increased (*p* < 0.05). Compared with UUO + exosome group, the level of IL‐6 in the UUO + exosomes + AH6809 group was significantly increased, and the level of IL‐10 was significantly decreased (*p* < 0.05) (Figure 4g).

## 4 DISCUSSION

Renal fibrosis has an important role in the development of CKD [2]. Inhibiting fibrosis or blocking the targets that trigger fibrosis can prevent the progression of CKD [18]. However, no simple animal model of renal fibrosis that can fully simulate human CKD has been developed. Previous studies have shown that the rodent unilateral ureteral obstruction (UUO) model can cause haemodynamic and metabonomic changes, leading to tubule damage, apoptosis, and necrosis, accompanied by macrophage infiltration and interstitial fibroblasts. The excessive proliferation and the transformation of myofibroblasts result in a large amount of extracellular matrix deposition, thereby forming renal fibrosis [19]. In this study, we used an UUO method to construct a mouse renal fibrosis model. Serum creatinine and urea nitrogen were highest at UUO0d and decreased at UUO14d and UUO21d. Similar trends were observed for the expression of PGE2. These data suggest that high levels of creatinine, urea nitrogen, and PEG2 expression reflect the acute stage of injury, while their decline may be related to fibrosis. In addition, previous studies have reported that low levels of PGE2 are associated with increased fibrosis in idiopathic pulmonary fibrosis [20]. Masson staining and Sirius red staining further revealed that the fibrosis degree gradually increased with the extension of UUO times until 14d. These data were consistent with the result of Atsuko et al. [21]. α‐SMA and FN are specific biomarkers of myofibroblasts [19]. In this study, we found that the expression of α‐SMA and FN increased with the extension of UUO times and then remained stable until day 14‐post modelling, suggesting that the transformation of myofibroblasts in UUO mice gradually increased until day 14. Moreover, compared with the control group, the expression of EP2 protein was significantly higher at 7 days after UUO (UUO7d group). In addition, compared with UUO7d group, UUO did not increase further on days 14 and 21, indicating that EP2 levels are related to the presence of fibrosis, and not necessarily to the degree of fibrosis.

### 4.1 Exosome from MSC activating EP2 receptor can alleviate renal fibrosis

Studies have shown that MSC exosomes play an important protective role in acute kidney injury and diabetic nephropathy [5, 19]. A recent study found that the culture supernatant of human umbilical cord blood MSCs has an inhibitory effect on renal fibrosis [17], suggesting that exosomes are the crucial active ingredient in the cell culture supernatant. In this study, we discovered that the BM‐MSC exosome might improve renal fibrosis and reduce M1 and M2 polarised macrophages in UUO mice.

PGE2 is the key to MSC‐mediated immune regulation [13]. Previous studies have shown that PGE2 plays an impotent role in antifibrosis [21]. However, so far, no studies have shown which receptor is involved in the regulation of MSC. Jensen et al. found that the EP2 agonist (butaprost) could reduce the TGF‐β‐induced FN expression and alleviate renal fibrosis [22]. Combining Jensen's data and our findings, we assumed that exosome from BM‐MSC activating EP2 receptor regulates the process of UUO renal fibrosis. Also, macrophage polarisation decrease caused by exosome and butaprost has an intrinsic relationship with the remission of renal fibrosis. Hong et al. found that inhibition of M1 and M2 macrophage polarisation reduced renal fibrosis [8].

Studies showed that bone marrow‐derived macrophages could transform to myofibroblasts through MMT. Their biopsies were examined for MMT cells that co‐express macrophage (CD68) and myofibroblast (α‐smooth muscle actin, α‐SMA) markers [23]. However, so far, no studies applied cell lines to research MMT. In our study, the mouse macrophage cell line RAW264.7 was cultured in vitro. Under the stimulation of LPS, the cell highly expressed the biomarker α‐SMA of myofibroblasts, indicating that cells can undergo MMT transformation in an inflammatory state, thereby increasing the number of myofibroblasts. In addition, the cell expresses the EP2 receptor, which can be used to study the PGE2/EP2 pathway. After stimulation with different concentrations of LPS, we found that there was no statistically significant difference in α‐SMA protein expression between 0.1 μg/ml and larger concentrations of LPS. Therefore, 0.1 μg/ml was selected as the optimal stimulation concentration. At the same time, we found that the expression of EP2 was consistent with the expression of α‐SMA, indicating that EP2 may participate in this process. We used different concentrations of the EP2 agonist butaprost, and the EP2 antagonist AH6809 interfered with LPS‐stimulated macrophages and found that Butaprost reduces the level of α‐SMA, and AH6809 increases the expression of α‐SMA; 20 μM was the optimal concentration. This suggested that activating the EP2 receptor could inhibit the transformation of macrophages into myofibroblasts while inhibiting the EP2 receptor can promote the transformation of macrophages myofibroblasts. Interestingly, exosomes from BM‐MSC have similar effects on macrophages with butaprost. Therefore we assume that exosome from BM‐MSC may inhibit the MMT by activating the EP2 receptor.

### 4.2 Exosome from MSC activating the EP2 receptor decreases the number of M1 and M2 macrophages and inhibits the release of the inflammatory factor from macrophages

Our results showed that exosome and butaprost both reduced the number of M1 (F4/80^+^CD86^+^) and M2 (F480/^+^CD206^+^) macrophages, inhibited the release of inflammatory factor IL‐6, and promoted the release of anti‐inflammatory factor IL‐10. However, these effects decreased on inhibition of the EP2 receptor. These data suggest that stem cell exosomes can activate EP2 receptors to inhibit the M1 and M2 macrophage polarisation and the secretion of inflammatory factors by macrophages. Furthermore, Wang et al. suggested that the majority of MMT cells in human and experimental renal allograft rejection co‐express the M2‐type macrophage marker CD206 [5]. Therefore, we assume that exosome and butaprost reduce MMT by inhibiting the polarisation of M2 and also reduce inflammation by inhibiting the polarisation of M1.

The decrease in the number of M2 macrophages and the increase in IL‐10 may be due to different subtypes of M2. According to literature, M2 macrophages are divided into three subtypes, M2a, M2b, and M2c. Among them, M2a and M2c overexpress CD206 and participate in tissue repair and fibrosis, while M2b secretes anti‐inflammatory factors such as IL‐10 and participates in immune regulation [6]. It is possible that exosomes activate EP2 receptors, regulate the transformation of macrophages into anti‐inflammatory phenotypes, reduce M1, M2a, and M2c as a whole, and increase M2b macrophages, thereby inhibiting renal fibrosis; however, this needs further investigation.

## 5 CONCLUSION

MSC exosomes can regulate the polarisation of M1 and M2 macrophages by activating the EP2 receptors, by inhibiting the secretion of pro‐inflammatory factors, and reducing myofibroblast transformation, thereby reducing extracellular matrix deposition and the formation of renal fibrosis. Nevertheless, the downstream pathway of EP2 needs to be examined in more detail since it has the potential to become a target for renal fibrosis treatment and can also inspire new ideas for clinical treatment of renal fibrosis.

## CONFLICT OF INTEREST

The authors have declared that no conflict of interest exists.

## PERMISSION TO REPRODUCE MATERIALS FROM OTHER SOURCES

None.

## Data Availability

The data that support the findings of this study are available from the corresponding author upon reasonable request.
